# Too much AKT turns PAX3-FKHR dead: A prospect of novel therapeutic strategy for alveolar rhabdomyosarcoma

**DOI:** 10.18632/oncotarget.713

**Published:** 2012-10-17

**Authors:** Mathivanan Jothi, Asoke K. Mal

**Affiliations:** Department of Cell Stress Biology, Roswell Park Cancer Institute, Buffalo, NY; Department of Cell Stress Biology, Roswell Park Cancer Institute, Buffalo, NY

Alveolar rhabdomyosarcoma (ARMS) is the aggressive subtype of muscle cancer in the pediatric population and children with ARMS have significantly poor prognosis and frequently present metastasis [1]. More than 80% of ARMS have chromosomal translocation t(2;13) generated novel fusion transcription factor PAX3-FKHR in which the DNA binding domain of PAX3 is fused to the carboxyl terminus of FKHR [1]. PAX3-FKHR protein acts as a potent transcriptional activator and influences expression of genes that ultimately contribute to its oncogenic behavior by modifying cell growth, migration, apoptosis, and muscle differentiation [1]. Fusion-positive PAX3-FKHR status in patients correlates with worse outcome and displays a more aggressive clinical phenotype that is consistent with cell lines and mouse models of ARMS [1]. Therefore, inhibiting PAX3-FKHR activity, a potential Achilles' heel, is a sound therapeutic strategy to eliminate ARMS cells that have benefit to children with this disease.

ARMS exhibit undifferentiated muscle lineage phenotype and resistance to terminal differentiation [1]. PAX3-FKHR activity sustains the undifferentiated state by suppressing differentiation, thus contributing ARMS tumorigenesis [1]. In this scenario, PAX3-FKHR exerts a negative regulation on key myogenic regulator MyoD-driven terminal differentiation in ARMS cells [1]. We investigated the molecular regulation of PAX3-FKHR activity to evade terminal differentiation in ARMS cells. Intriguingly, we discover that activated AKT status in ARMS cells balance the transcriptional activity of PAX3-FKHR via phosphorylation. In particular, we find that attenuated AKT activity sustains PAX3-FKHR activity, however, acute AKT activation blocks PAX3-FKHR in gene activation including its bona fide target MyoD expression, preventing ARMS cell from escaping differentiation. However, acute AKT activation along with ectopic MyoD expression permits ARMS cells to undergo terminal differentiation. We recently published this finding in Cell Cycle Journal [2]. AKT downstream effector GSK mediated phosphorylation of PAX3-FKHR increases its transcriptional activity and is inhibited by GSK specific inhibitors [3], raising the possibility that AKT mediated inhibition of PAX3-FKHR activity either directly or via GSK. PAX3-FKHR retains two consensus AKT phosphorylation sites in the FKHR domain [2]. Mutation of the AKT sites in PAX3-FKHR results in increased transcriptional activity (personal communication), strongly suggesting that AKT directly regulating PAX3-FKHR activity. Activation of AKT acts as a promyogenic signal for terminal differentiation (Fig. 1). Conversely, AKT activation has been linked to both oncogenesis and poor prognosis and consequently AKT inhibitors that limit AKT signals are being developed as cancer therapeutics [4]. However, inhibiting AKT increases cancer cell motility and invasion abilities that are important for tumor metastasis. Indeed, expression of activated AKT1 in ErbB2 background enhances tumorigenesis, but shows fewer metastatic lesions compared with control mice [5], indicating that AKT1 functions as a suppressor of tumor metastasis. The anti-metastatic function of AKT was also reported in breast cancer cells [4]. Moreover, AKT allosteric inhibitor MK-2206 showed limited *in vivo* activity against solid tumor and ALL xenografts [6]. Thus, there are pressing concerns of inhibiting AKT for therapeutic benefit in some cancers.

**Figure 1:**
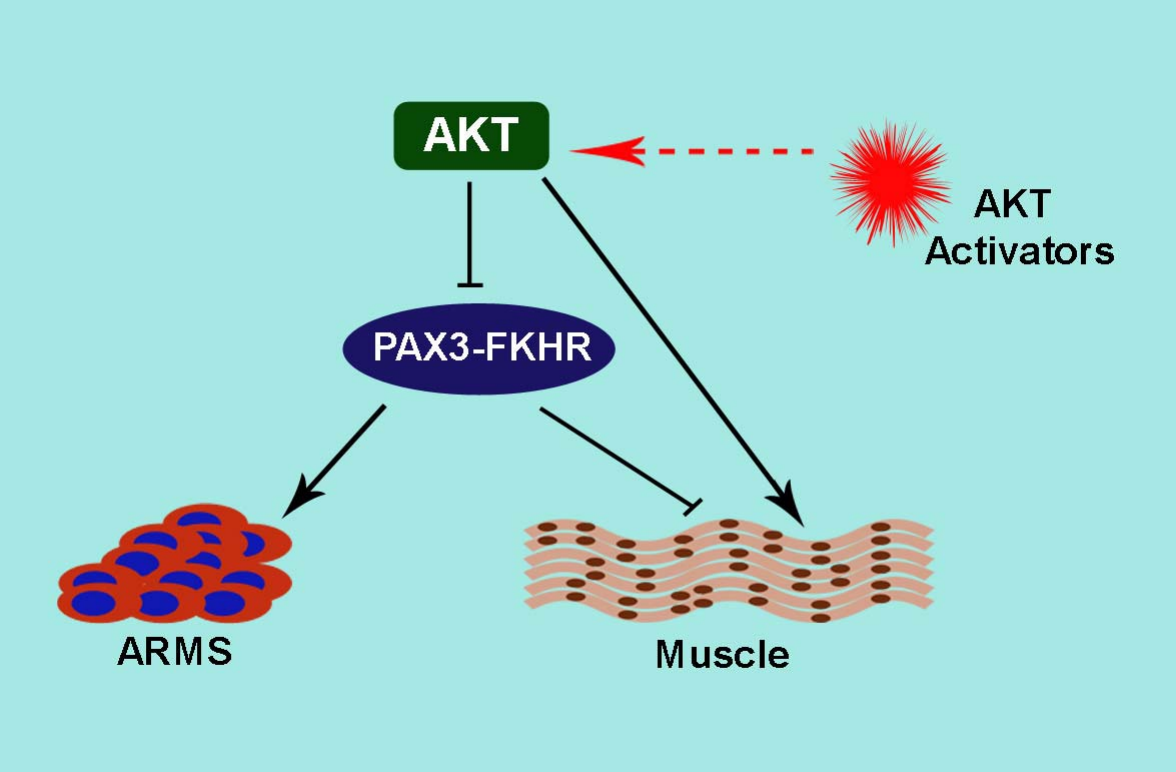
This model represents the action of AKT on PAX3-FKHR activity in ARMS and terminal muscle differentiation PAX3-FKHR plays a dynamic role in ARMS oncogenesis and terminal differentiation block. Enhanced AKT activation inhibits PAX3-FKHR activity and entails for terminal differentiation. Elevating AKT activity by specific small molecule AKT activators may be exploited to inactivate PAX3-FKHR, leading to foil ARMS.

Our study revealed the unexpected finding that elevating AKT activity can inactivate PAX3-FKHR in ARMS cells [2]. This is important because it shows that acute activation of AKT inactivates PAX3-FKHR activity known to be involved in development and metastasis of ARMS. This new discovery poses a host of new questions and challenges that activator-induced AKT activation may hinder PAX3-FKHR activity, thus offer a therapeutic benefit to ARMS. Dilling et al. showed that cell lines derived from childhood ARMS are very sensitive to the growth-inhibitory effects of the rapamycin [7]. Rapamycin induced feedback activation of AKT is well documented [8]. Therefore, sensitivity of ARMS cells to rapamycin may result in AKT activation induced inhibition of PAX3-FKHR activity. In a similar way, activation of AKT may provide a therapeutic benefit in other cancers. In this case, we may envision the potential benefit of AKT activation by TNF-α, which is used in the regional treatment of locally advanced soft tissue sarcomas, metastatic melanomas and other unresectable tumors [9]. Constitutive PI3K/AKT activation in AML patients showed a better prognosis [10], indicating a potential function of AKT activation in clinical success of cancer treatment. A characteristic abnormality of ARMS cells is a blockade of terminal differentiation. A strategy of inducing ARMS cells to differentiate, termed ‘differentiation therapy’ is an elegant alternative that could limit the current toxic therapeutic approach. The bi-transgenic AKT1 and ErbB-2 developed mammary tumors exhibit more differentiated phenotypes than ErbB2 alone [5]. This study suggests that AKT could promote tumor differentiation in certain setting. PAX3-FKHR abolition in ARMS cells led to terminal differentiation [1], therefore, it is worth considering the potential role of AKT activation-dependent inactivation of PAX3-FKHR in this effort (Fig. 1). Taken together, we venture that acute AKT activation should be evaluated for therapeutic benefit in ARMS and also in certain types of tumor.

## References

[1] De Giovanni C, Landuzzi L, Nicoletti G (2009). Future Oncol.

[2] Jothi M, Nishijo K, Keller C (2012). Cell Cycle.

[3] Zeng FY, Dong H, Cui J (2010). Biochemical and biophysical research communications.

[4] Toker A, Yoeli-Lerner M (2006). Cancer research.

[5] Hutchinson JN, Jin J, Cardiff RD (2004). Cancer research.

[6] Gorlick R, Maris JM, Houghton PJ (2012). Pediatric blood & cancer.

[7] Dilling MB, Dias P, Shapiro DN (1994). Cancer research.

[8] Wan X, Harkavy B, Shen N (2007). Oncogene.

[9] van Horssen R, Ten Hagen TL, Eggermont AM (2006). The oncologist.

[10] Tamburini J, Elie C, Bardet V (2007). Blood.

